# Evaluation of the Antibacterial Activities of Mangrove Honeybee Propolis Extract and the Identification of Transpeptidase and Transglycosylase as Targets for New Antibiotics Using Molecular Docking

**DOI:** 10.3390/antibiotics12071197

**Published:** 2023-07-17

**Authors:** Maha A. Alshiekheid

**Affiliations:** Department of Botany and Microbiology, College of Science, King Saud University, Riyadh 11451, Saudi Arabia; malsheikh@ksu.edu.sa

**Keywords:** *Apis mellifera*, mangrove honeybee propolis, *Avicennia marina*, Saudi Arabia, Arabian Gulf, antibiotic resistance, cell wall enzymes

## Abstract

Developing new antibiotics is a critical area of research that grows as a result of the increasing problem of antibiotic resistance. Scientists search for new antibiotics by screening natural sources such as soil, plants, and marine environments. One of the iconic plants in the marine environment is the mangrove, which is a source of honeybee propolis. Propolis collected from the grey mangrove *Avicennia marina* on Tarout Island, the Eastern Province of Saudi Arabia, was used to evaluate antibacterial activities against three pathogenic bacteria: gram-negative *Enterobacter cloacae* (RCMB 001(1) ATCC^®^ 23355^TM^), gram-positive methicillin-resistant *Staphylococcus aureus* (clinical isolate), and *Streptococcus mutans* Clark (RCMB 017(1) ATCC^®^ 25175^TM^). The results indicate the effectiveness of the methanolic extract of such propolis. The chemical composition of this extract was analyzed using LC-MS, and four compounds were identified (alginic acid, carrageenan, fucoxanthin, cycloeudesmol). Their modes of action were evaluated against bacterial cell walls. Bacterial transpeptidase and transglycosylase on the surface are basic for cell divider amalgamation, and numerous antimicrobials have been created to target these compounds. Molecular docking was employed to predict the interactions of four compounds and *S. aureus* to predict interaction. Alginic acid was found to be the best interaction with a score of −7.44 Kcal/mol with distance ranges between 2.86 and 3.64 and RMSD refined below 2 Å. Carrageenan with −6.64 Kcal/mol and a distance of 3.05 and 2.87 came second. Then, fucoxanthin with −6.57 Kcal/mol and a distance of 1.4. Finally, cycloeudesmol with a score of −4.6 Kcal/mol and a distance of 2.87 showed the least activity. The first three compounds interacted effectively and could form very promising chemicals that could be used one day against pathogenic bacteria in the future.

## 1. Introduction

Antibiotic resistance is an increasing problem around the world, making it difficult, if not impossible, to treat infections that were previously easily treated with antibiotics [1,2,3]. This can result in prolonged hospital stays, increased healthcare costs, and even mortality in some situations [4]. Antibiotic overuse and misuse are key contributors to the development of antibiotic resistance [5]. When antibiotics are overused or misused, bacteria have a greater opportunity to evolve resistance. This can occur when antibiotics are prescribed for viral infections that are not treatable with antibiotics, or when people do not finish the entire course of antibiotics prescribed to them [6,7]. Antibiotic resistance can be prevented by using antibiotics responsibly, improving infection prevention, and developing novel medications [8,9,10].

For decades, bee products have been utilized medicinally, and current studies have focused on their potential as a source of new antibiotics [11,12]. Honey, for example, has a variety of bioactive chemicals with antibacterial effects, including hydrogen peroxide, methylglyoxal, and flavonoids [13]. These chemicals are hypothesized to cause cell death by disrupting bacterial cell membranes and interfering with metabolic processes [14]. Another bee product is propolis, which is a resinous substance collected by bees to seal crevices in their colonies. Honeybees, *Apis mellifera*, gather propolis, a sticky material, from the buds and bark of trees and other plants. Bees utilize it to guard against infections like germs and viruses [15,16,17,18,19]. Propolis has been used as a medicine for centuries, and it has been found to have a number of physiologically active ingredients. Propolis comprises a range of chemicals, although its precise composition might vary depending on the area and plant sources from which it is harvested. It has antimicrobial activity against a wide variety of bacteria and fungi, including some antibiotic-resistant species [20]. Propolis contains a number of bioactive components, including flavonoids, which are plant pigments with recognized antioxidant effects that may assist decreasing inflammation and strengthening the immune system. Phenolic acids are organic acids that are also found in many fruits and vegetables. They have been shown to have anti-inflammatory, antioxidant, and antimicrobial properties. Terpenes are kinds of propolis that contain trace levels of essential oils, which are volatile molecules with therapeutic effects. All of these compounds have antibacterial effects [21,22].

Mangrove honeybee propolis is made by bees that feed on the nectar and pollen of mangrove trees [23]. Mangrove trees grow in coastal areas and estuaries, and their honeybee propolis have been studied for their medicinal effects [24]. Mangrove honeybee propolis of the Brazilian mangrove (*Baccharis dracunculifolia*) has been demonstrated in studies to have antibacterial, anti-inflammatory, and antioxidant characteristics, making it a prospective option for application in the development of new treatments and therapies [25]. The inclusion of flavonoids, phenolic acids, and terpenes in mangrove honeybee propolis is assumed to be responsible for its antibacterial effect, which has been found to suppress the growth of a variety of bacteria and fungi, including some antibiotic-resistant species. The effect of chemical compounds that are found in propolis on microbes could be due to their impact on bacterial structure and multiplication [26].

Enzymes such as transglycosylase (TG) and transpeptidase (TP) are crucial for the creation and upkeep of the bacterial cell wall. The intricately designed bacterial cell wall surrounds and defends the bacterial cell and is crucial for bacterial survival and development [27]. Most antimicrobial chemicals such as β-lactams (e.g., penicillin and methicillin), glycopeptides (e.g., vancomycin and teicoplanin), and glycolipopeptides restrain the action of TP [28]. Antibiotics such as penicillin target both TG and TP, interfering with their enzymatic activity and preventing appropriate production of the bacterial cell wall. Bacteria become more vulnerable to osmotic pressure and are more prone to lyse as a result [29].

The study of the medical application of propolis is very scarce throughout the Middle East, especially propolis collected from Arabian Gulf mangroves [30]. So, the aim of this work is to evaluate the antibacterial activity of mangrove propolis extraction against some multidrug-resistant (MDR) bacteria, chemically analyze the mangrove propolis, and try to assess how some of the isolated chemical compounds work against bacterial cell walls, especially transglycosylase (TG) and transpeptidase (TP) enzymes using molecular docking.

## 2. Results

### 2.1. Evaluating the Antibacterial Activity of Mangrove Honeybee Propolis Extract

**Figure 1:**
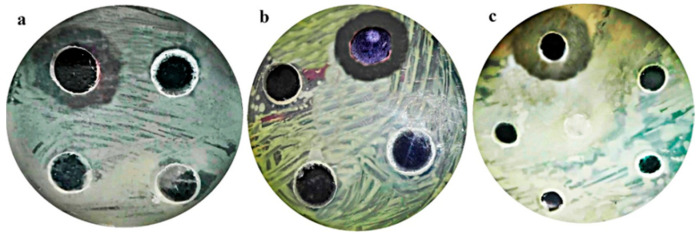
Antibacterial activity of mangrove honeybee propolis extract against (**a**) *Enterobacter cloacae*, (**b**) *Streptococcus mutans*, and (**c**) *Staphylococcus aureus*.

It was discovered that mangrove propolis extract provided an inhibitory impact against the tested pathogens when its antibacterial activity was assessed against a panel of chosen human pathogens. Table 1 and Figure 1 list the determined inhibition zones. The *Staphylococcus aureus* was chosen for further investigation as it was the most affected pathogen with an inhibition zone of 35 mm.

### 2.2. Ultrastructural Changes of the Tested Staphylococcus aureus

**Figure 2:**
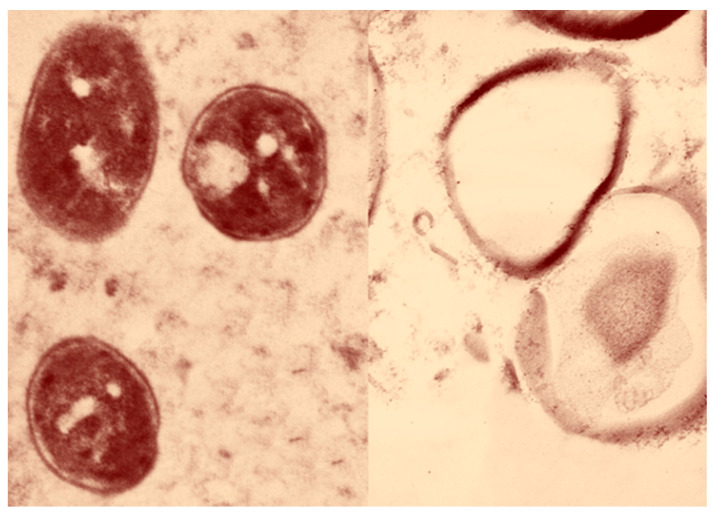
TEM micrograph of *Staphylococcus aureus* cells. The normal cells appear on the left side and the treated cells appear on the right side with the ruptured cell wall and leakage of cytoplasmic components.

Regarding the *Staphylococcus aureus* TEM-stained ultrathin slices (70 nm), cells that had not been treated appear in normal conditions including a spherical form, a hard surface covered in cytoplasm, constant tight contact with the cell wall, and a normally intact cytoplasmic membrane. After treatment with mangrove propolis extract, complete cellular destruction was seen along with breaches in the cell wall and leakage of cytoplasmic components. This led to membrane damage that had the appearance of being ready to rupture, leakage of intracellular components, and cellular damage that eventually ended in total cell deformation (Figure 2).

### 2.3. LC-MS Analysis of Propolis Methanolic Extracts

**Figure 3:**
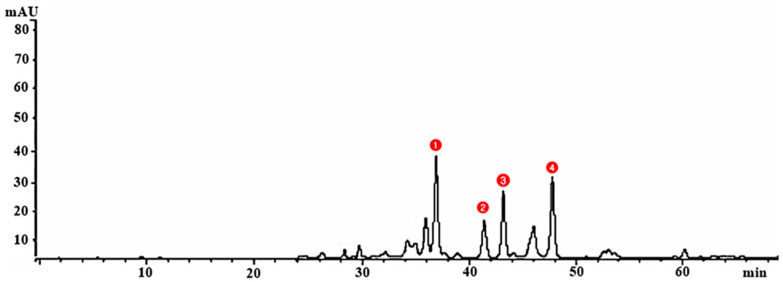
The LC-MS chromatogram for the mangrove honeybee propolis methanolic extract the four considered compounds were 1. alginic acid, 2. carrageenan, 3. fucoxanthin, and 4. cycloeudesmol.

Utilizing LC-MS to examine the mangrove honeybee propolis methanolic extracts, four active substances were discovered: alginic acid, carrageenan, fucoxanthin, and cycloeudesmol. The first and most abundant compound was alginic acid at a retention time of 37 min., and the last compound considered was cycloeudesmol at a retention time of 48 min. Carrageenan and fucoxanthin were also identified in Figure 3.

### 2.4. Molecular Docking of the Mangrove honeybee Propolis Extracted Compounds

**Figure 4:**
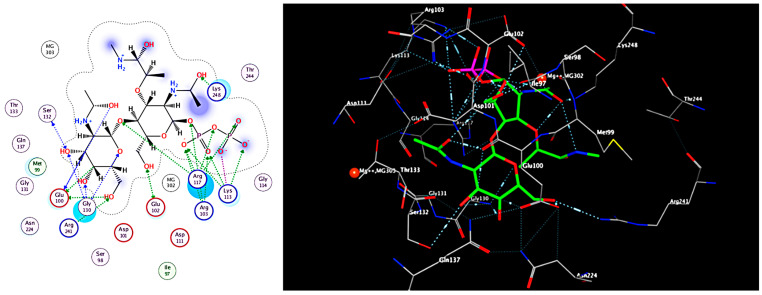
Interaction of the reference ligand (lipid II analog) with the active site of (SamGT) in 2D and 3D.

**Figure 5:**
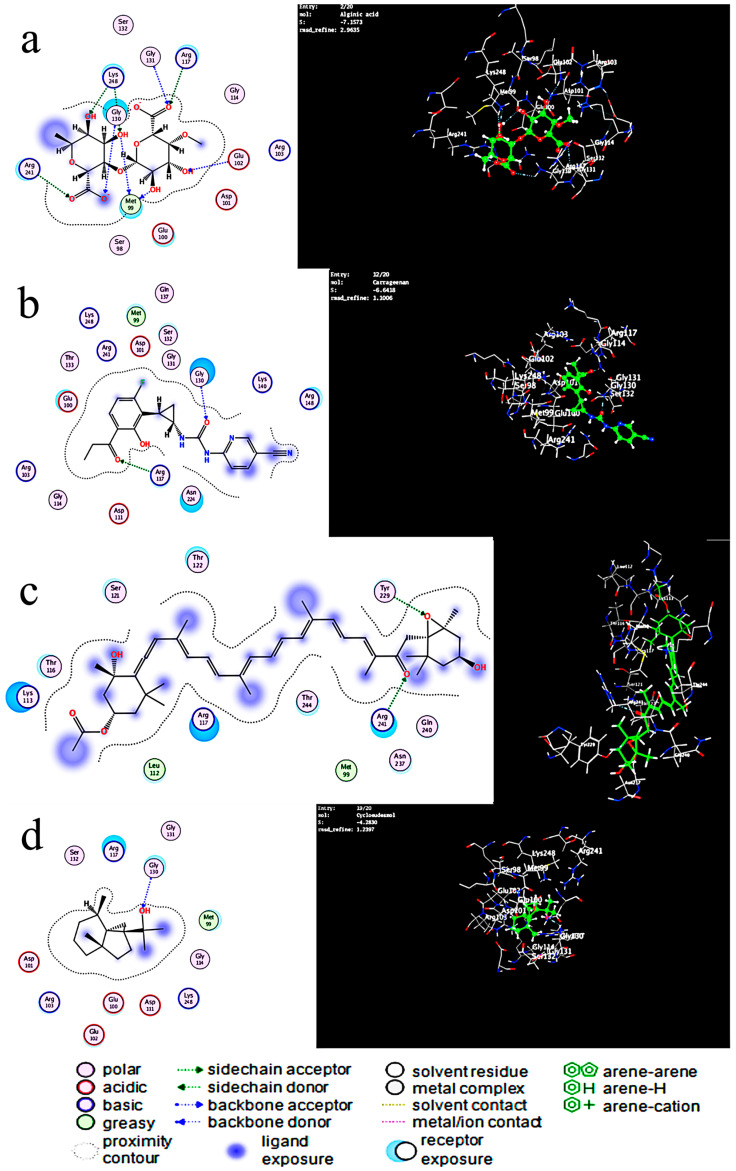
(**a**) Interaction of compound 1 (alginic acid) with the active site of (SamGT) in 2D and 3D; (**b**) interaction of compound 2 (carrageenan) with the active site of (SamGT) in 2D and 3D; (**c**) interaction of compound 3 (fucoxanthin) with the active site of (SamGT) in 2D and 3D; (**d**) interaction of compound 4 (cycloeudesmol) with the active site of (SamGT) in 2D and 3D.

In Table 2, the lowest energy needed for interaction was observed in the alginic acid interaction with SamGT with a score of −7.44 Kcal/mol and RMSD refined below 2 Å with six H-acceptor interactions with six amino acids and six ionics with three amino acids, which are displayed in the table. Carrageenan formed two H-acceptor interactions with a score of −6.64 Kcal/mol. Fucoxanthin, on the other hand, had a score of −6.57 Kcal/mol, forming one H-acceptor interaction with one amino acid, while cycloeudesmol had a score of −4.28 Kcal/mol, forming one H-acceptor interaction with an amino acid in Figure 4 and Figure 5.

## 3. Discussion

A significant public health issue known as antibiotic resistance is brought on when bacteria learn to withstand the effects of antibiotics [31]. Antibiotic resistance can have serious consequences, including longer hospital stays, higher medical costs, and increased mortality rates. Infections produced by antibiotic-resistant bacteria may necessitate more intrusive treatments, such as surgery, and may even result in death in some situations [32]. For decades, nature has provided a rich source of antibiotics, and many of today’s routinely used antibiotics are derived from natural substances [33]. Today, scientists are looking for new antibiotics in a variety of natural sources to overcome antibiotic resistance. These sources include soil, plants, marine ecosystems, and even insects [34]. Screening extracts or isolated chemicals from various sources for their capacity to prevent the growth of bacterial strains is one of the techniques used for identifying novel antibiotics from nature. Traditional procedures such as agar diffusion experiments may be used [35].

Due to its antibacterial and anti-inflammatory qualities, honeybee products have been utilized for therapeutic purposes for ages [36]. Now, they are also a target for several studies for generating new antibacterial agents [37]. One of these products is propolis, the resinous substance that is collected by honeybees from plant sources to protect their hive against pathogens. The plant source of the propolis usually plays a key role in determining its beneficial effects [30]. Mangrove honeybee propolis is renowned for having a special chemical makeup that contains a variety of bioactive substances with antibacterial, anti-inflammatory, and antioxidant activities [26].

The present work forms the first work that evaluates the antibacterial activities of Arabian Gulf mangrove honeybee propolis. The generated results appear compatible with the previous studies that discussed the biological activities of bee propolis, including solitary wild bees, such as *Scaptotrigona aff. postica* from Brazil, which also collected propolis from mangroves [38]. The results indicate high activities against the three tested pathogens (*Enterobacter cloacae*, a member of the normal gut flora, but some strains have been linked to respiratory and urinary tract infections in people with impaired immune systems. Also, numerous outbreaks of infections caused by *Enterobacter cloacae* have been documented in newborn facilities, where it has become an important nosocomial pathogen [38]. *Streptococcus mutans* is the primary cause of oral infections and tooth deterioration [39]. *Staphylococcus aureus* is a typical resident of human and animal skin but is occasionally capable of inflicting infections on many organs and implicated in food poisoning. It is one of the most serious problems for public health, as most of its strains developed antibiotic resistance against a panel of very effective antibiotics in the market [40]). Three tested bacteria were chosen to cover external, internal, and buccal cavity pathogens that show resistance against several available antibiotics.

*Staphylococcus aureus* shows very high sensitivity toward mangrove honeybee propolis extract, and the TEM results show how the extract affects the cell wall of this bacteria leading to its death. Zhang et. al. found that red propolis collected by the honeybee *Apis mellifera* from a Chinese mangrove has great antibacterial activity against *Staphylococcus aureus*, which agrees with our result. However, there were different effective isolated compounds identified, including pinobanksin, pinobanksin-3-acetate, and chrysin, which were bioactive compounds [41]. The Indonesian mangrove *Sonneratia caseolaris* (ethanolic extract of leaves) showed high activity against *Staphylococcus aureus* [42]. Such data indicate how mangroves and their products could be very important sources.

The present results encourage further investigation of the chemical composition of mangrove honeybee propolis extract and modeling the way by which any of these chemicals alter cell wall biology. The four chemical compounds identified in this study were new for honeybee propolis compared to any previously studied propolis [29,41,42]. This may be due to the nature of the Arabian Gulf and the algal community associated with mangroves in the study area. Compounds such as alginic acid and fucoxanthin are very common chemicals in brown and red algae [43,44,45]. The analysis of alginic acid interaction with two enzymes found on the bacterial cell wall (transglycosylase and transpeptidase) may reveal the mode of action of the antibacterial activities of this compound.

Antibiotics like penicillin and cephalosporins target both transglycosylase and transpeptidase, which inhibits their action and prevents the creation and upkeep of the bacterial cell wall. In the end, the bacteria are killed by cell lysis. To counteract the effects of antibiotics, certain bacteria have created enzymes like lactamases, which cleave the antibiotics’ lactam rings and render them inactive. As a result, germs that are resistant to antibiotics have started to appear and present a serious threat to public health nowadays [46].

Based on the result of docking the best fit, which has a score of (−7.447 Kcal/mol) between the ligand and the receptor and the RMSD refined below 2 Å, which is good, is the alginic acid in Figure 5a where six H-acceptor interactions formed Arg 117 (connected to an oxygen atom of the ligand): two with Lys248 (binds to the N-acetyl, which connects to the hydroxyl group of the ligand), one with Arg 241, one with Gly130, and one with Glu102. Alginic acid also formed six ionic bonds (two with Gly 130, two with Arg241, two with Arg117) amino acids, and all of these amino acids are crucial amino acids in distances ranging from 2.86 to 3.64. In the reference ligand, the interaction gave a score of −6.8 Kcal/mol and involved six hydrogen donor interactions: four with Glu100, one with Glu102, and one with Ser132, and it formed sixteen hydrogen acceptor interaction: three interactions with Arg103, three interactions with Glu130, one with Ser132, one with Arg241, one with Glu102, five with Arg117, and one with Lys248, and there are four ionic bonds with Arg117 and three interactions with Arg103. Arg241 also shapes the salt bridge with Glu100. Arg117 and Arg103 relate to the pyrophosphate of lipid II analog 3, and Arg117 moreover connects with the β1–4 glycosidic oxygen of lipid II analog 3. The Mg2+ molecule connects to Glu102, and the spine carbonyl, as seen in Figure 4, and the distance between atoms ranges from 2.70 to 3.86.

The O5 of carrageenan in Figure 5b formed an H-acceptor interaction with Arg117 (connected to an oxygen atom of the ligand) at distance of 3.05, and the O4 of carrageenan made an H-acceptor interaction with Gly 130 with a score of (−6.64 Kcal/mol) with an RMSD refined below 2 Å, which is good considering the second effective at distance of 2.87. The O5 of fucoxanthin in Figure 5c formed one H-acceptor interaction with Arg 241 with a score of (−6.57 Kcal/mole) with an RMSD refined below 2 and a distance of 1.4. Cycloeudesmol in Figure 5d is not effective at all, as it scored a high value compared to the others, which is a bad interaction value, and the O1 of cycloeudesmol formed an H-acceptor interaction with Arg117 at a distance of 2.87 and a score of −4.6 Kcal/mol.

The smaller value of the binding affinity means that the energy needed for drug-receptor interaction is small; the drug-receptor bond is more stable and will increase the predicted activity. The higher number of hydrogen bonds indicated that the compound is more active. Considering the previous results, I have concluded that we have three compounds: alginic acid, carrageenan, and fucoxanthin, which can be further investigated in clinical trials.

Mangrove honeybee propolis is promising as a possible source of organic substances with medicinal effects. Honeybee products as a whole have been promised as a novel source of antibiotics; further investigation is required to fully grasp their potential and turn them into medicines that are therapeutically effective. The sustainability of honeybee populations and adherence to moral standards in the manufacturing and consumption of honeybee products are also crucial.

## 4. Materials and Methods

### 4.1. Propolis Sample Collection

The propolis samples were collected from honeybee (*Apis mellifera*) hives during the flowering season (from February to June) of grey mangrove *Avicennia marina* on Tarout Island, the Eastern Province of Saudi Arabia (26.59286 N, 50.057994 E). A total of 10 beehives were established near a large mangrove forest (larger than 2000 km^2^) for collecting propolis for scientific research. The confirmation of the source of propolis was performed using a pollen analysis test, and the pollen grains of *Avicennia marina* were clearly identified in Figure S1. By soaking the propolis pieces in methanol for 48 h and then separating the methanolic extract from the residue by filtering, the active chemicals found in the propolis samples were recovered. A portion of each extraction was used for chemical analysis and antimicrobial activity evaluation after the extract had dried. Dimethyl sulfoxide (DMSO) was then used to dissolve the powdered propolis extract [30].

### 4.2. Antibacterial Activity of the Mangrove Honeybee Propolis Extract

MDR bacteria and fungi were obtained from the Bioproducts Research Chair, College of Science, King Saud University, Riyadh, Saudi Arabia. Gram-negative bacteria included *Enterobacter cloacae* (RCMB 001(1) ATCC^®^ 23355^TM^), whereas gram-positive bacteria included methicillin-resistant *Staphylococcus aureus* (clinical isolate) and *Streptococcus mutans* Clark (RCMB 017(1) ATCC^®^ 25175^TM^). The purified colonies of bacteria used for the test were cultured on a nutrient broth medium, which was made by adding 13 gm of a bovine extract–peptide–sodium chloride mixture to 1 L of distilled water, mixing until completely dissolved, and sterilizing by autoclaving at 121 °C for 15 min. After incubation at 37 °C for 24 h in a shaker incubator, testing was conducted [47]. The antibacterial efficacy of mangrove honey and propolis extract was evaluated using the agar well diffusion method (using nutritional agar media for testing microorganisms). After that, the plates were tested for human pathogenic bacteria for 24 h at 37 °C. Using a clear ruler, the zones of inhibition of harmful bacteria were measured. Each test was performed three times for each pathogenic species that was considered [48].

### 4.3. Transmission Electron Microscopy (TEM)

TEM (JEOL 1010) was used to show the morphological changes in *Staphylococcus aureus* after being exposed to mangrove honeybee propolis extract. The samples were fixed in 3% glutaraldehyde for TEM preparation, washed in a phosphate buffer, and then post-fixed for five minutes at room temperature in a potassium permanganate solution. The samples were dehydrated for 15 min in each ethanol dilution, ranging from 30% to 90%, and then for 30 min in absolute ethanol. Through a graded series of injections of epoxy resin and acetone, the samples were finally immersed in pure resin. On copper grids, extremely thin pieces were gathered. After that, sections were double stained in lead citrate and uranyl acetate using a JEOL-JEM 1010 transmission electron microscope at 70 kV, transmission electron microscopy unit, College of Science, King Saud University, Riyadh, Saudi Arabia [49,50]. The final image was colored using PhotoScape 3.7.

### 4.4. Chemical Analysis of Mangrove Honeybee Propolis Extract

LC-MS (Liquid Chromatography–Mass Spectrometry) is a powerful analytical technique that combines the separation capabilities of liquid chromatography with the detection and identification capabilities of mass spectrometry. Either a solution or preabsorption on the packing material can be used to introduce the sample for chromatography. It has been determined that the latter technique is preferable, particularly for plant extracts and plant-origin materials, such as propolis, which are frequently only partially soluble in the initial eluent [30]. By combining silica gel (approximately 1–2 g for every 1 g of sample) with an appropriate low boiling point solvent (such as dichloromethane), the sample is preabsorbed. The mixture was then dried and allowed to move freely inside the flask after the solvent was evaporated using a rotary evaporator. By putting the flask under a strong vacuum for 15 to 30 min, the remaining traces of the solvent were eliminated. Obtaining a thoroughly dry and easily flowing mixture is crucial [51].

The LC/MS analysis was performed with a Dionex 3000 UHPLC pump linked to a Thermo Fisher Scientific (Bremen, Germany) QExactive (Orbitrap) mass spectrometer. Prior to LC-MS, crude samples and purified chemicals were produced in methanol at 1 mg/mL. A reverse-phase 5 m C18 column (4.6150 mm) (Hypersil, Thermo) was employed, and the elution was performed using a gradient at a flow rate of 0.3 mL/min, with the mobile phase consisting of 0.1% *v*/*v* formic acid in water and 0.1% *v*/*v* formic acid in acetonitrile (the A and B solvents). The identification of [M − H]^−^ was made possible by the ESI interface in negative ionization. The capillary and cone spray voltages were 4.0 kV and 35 V, respectively [52].

### 4.5. Molecular Modelling Study

**Figure 6:**
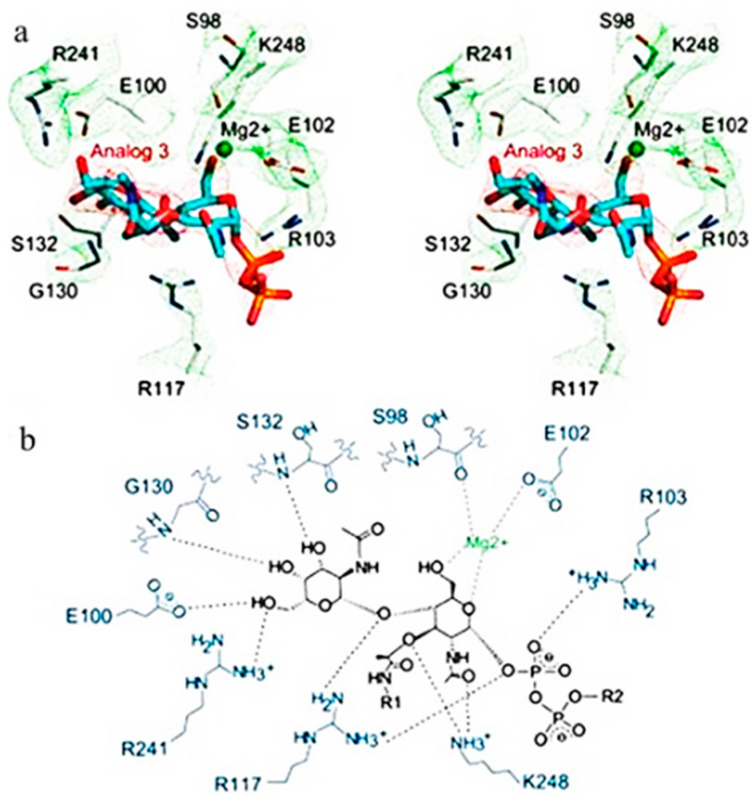
Stereo and schematic views of the interaction between SaMGT and the lipid II analog (**a**) 3D and (**b**) 2D.

**Figure 7:**
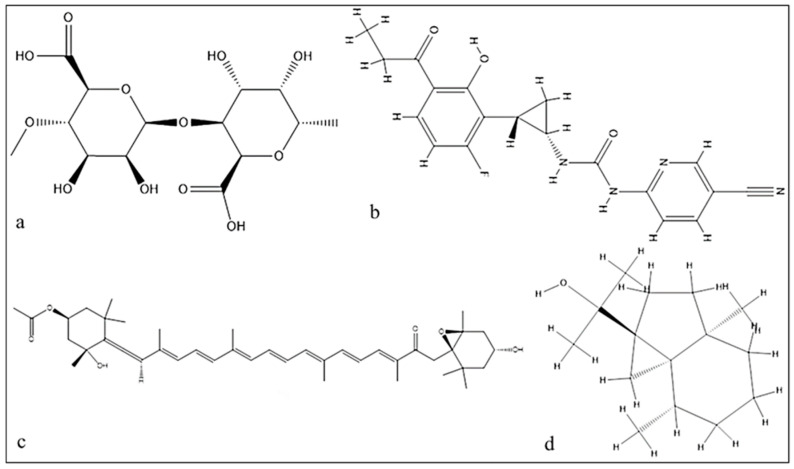
Chemical structure of the tested compounds: (**a**) alginic acid; (**b**) carrageenan; (**c**) fucoxanthin; (**d**) cycloeudesmol.

Molecular modeling studies have been initiated to support the putative mechanism of the action of the compounds identified from the tested mangrove honeybee propolis extract and optimize a reliable model for predicting new potent hits. The program used is for drawing the ligands was Chemdraw 20.0 (CambridgeSoft) (Perkin Inc., Waltham, MA, USA), and we used the wave function spartan v 14.0 program for minimizing energy. Then, the X-ray crystal structure of the crystal structure of *Staphylococcus aureus* membrane receptor transglycosylase Pdb id: (3VMT) from the protein data bank was obtained [53]. A docking study was carried out for the target compounds into *S. aureus* using Moe software 2015 [54]. The same software was used to visualize the binding modes/interactions of the tested compounds and the ligand (lipid II analog) in Figure 6 and Figure 7.

Molecular docking simulation studies have revealed that the key residues in the major hinge regions are Glu100, Ser132, Arg241, Arg117, Gly130, Lys248, Arg103, and Glu102. These are believed to be important for explaining the binding behavior of the lipid II analog with the *S. aureus* membrane, as reported [53].

Glu100 within the acceptor location was proposed to act as a common base for the 4-OH of GlcNAc to encourage the transglycosylation response. Also, the lipid II-analog authoritative stash of *S. aureus* MGT (referred to as SaMGT), Glu100, Ser132, and Arg241 tie to the GalNAc buildup, in which Ser132 tie to the GalNAc through spine amide bunch (Figure 1B). The Arg 241 shapes the salt bridge with Glu100 as seen within the structure of *S. aureus* PBP2. Glu100 in the acceptor site acts as a general base for the 4-OH of GlcNAc to facilitate the transglycosylation reaction (Huang et al.). Arg117 and Arg103 are connected with the pyrophosphate of lipid II analog 3, and Arg117 moreover interatomic with the β1–4 glycosidic oxygen of lipid II analog 3. Surprisingly, the d-lactyl ether moiety of MurNAc, which is part of the pentapeptide and assumed to take an interest within the transpeptidation response [27], connects with Lys 248 within the TG space of SaMGT; additionally, Lys 248 binds to the N-acetyl gather of MurNAc and 1 Mg^2+^ ties Glu102 and the spine carbonyl [55].

## Figures and Tables

**Table 1:** Antibacterial activity of mangrove honeybee propolis extract against multidrug-resistant (MDR) gram-positive and gram-negative bacteria measured by inhibition zone (mm).

Growth Inhibition Zone (mm)		
*Enterobacter cloacae*	*Streptococcus mutans*	*Staphylococcus aureus*
20 ± 0.2	18 ± 0.2	35 ± 0.2

The numbers represent means ± standard deviations.

**Table 2:** Results of molecular docking showing (a) binding-free energy (kcal/mol). (b) Amino acids indicated by the asterisk are involved in the bonds with the ligands. (c) Root-mean-square resemblance between the original ligand and the new ones (should be below 2 Å).

Compound	(a) Binding Energy (Score) (E)	(b) Amino Acids Involved in the Receptor	(c) RMSD Refine Å	Interaction
Alginic Acid	−7.447	Arg241, Arg117, Gly130, Lys248, and Glu102	1.22	H acceptor, ionic
Carrageenan	−6.64	Gly130-Gly117	1.1	H acceptor
Fucoxanthin	−6.57	Arg241	1.75	H-acceptor
Cycloeudesmol	−4.6	Arg117	1.55	H-acceptor
(Ref. Ligand) (Lipid II Analog)	−6.8	Arg241, Arg 103, Glu100, Lys248, Glu102, Ser132, Gly130, and Arg117	1.55	H donor, H acceptor, ionic

## Data Availability

All data were provided in the manuscript.

## Supplementary Materials

The following supporting information can be downloaded at https://www.mdpi.com/article/10.3390/antibiotics12071197/s1, Figure S1: Pollen grain of *Avicennia marina* from test propolis samples.
