# Supplementary material for: Evaluation of the Antibacterial Activities of Mangrove Honeybee Propolis Extract and the Identification of Transpeptidase and Transglycosylase as Targets for New Antibiotics Using Molecular Docking
Source: Antibiotics (Basel). 2023 Jul 17;12(7):1197. doi: 10.3390/antibiotics12071197 (PMC10376730; doi:10.3390/antibiotics12071197)

Figure S1. The pollen grain of *Avicennia marina* identified from under test propolis according the following reference:

-Suc, Jean-Pierre, Séverine Fauquette, and Speranta-Maria Popescu. "L'investigation palynologique du Cénozoïque passe par les herbiers." In Actes du Colloque "Les herbiers: un outil d'avenir. Tradition et modernité", Villeurbanne. Edit. Association française pour la Conservation des Espèces Végétales, Nancy, pp. 67-87. 2004.

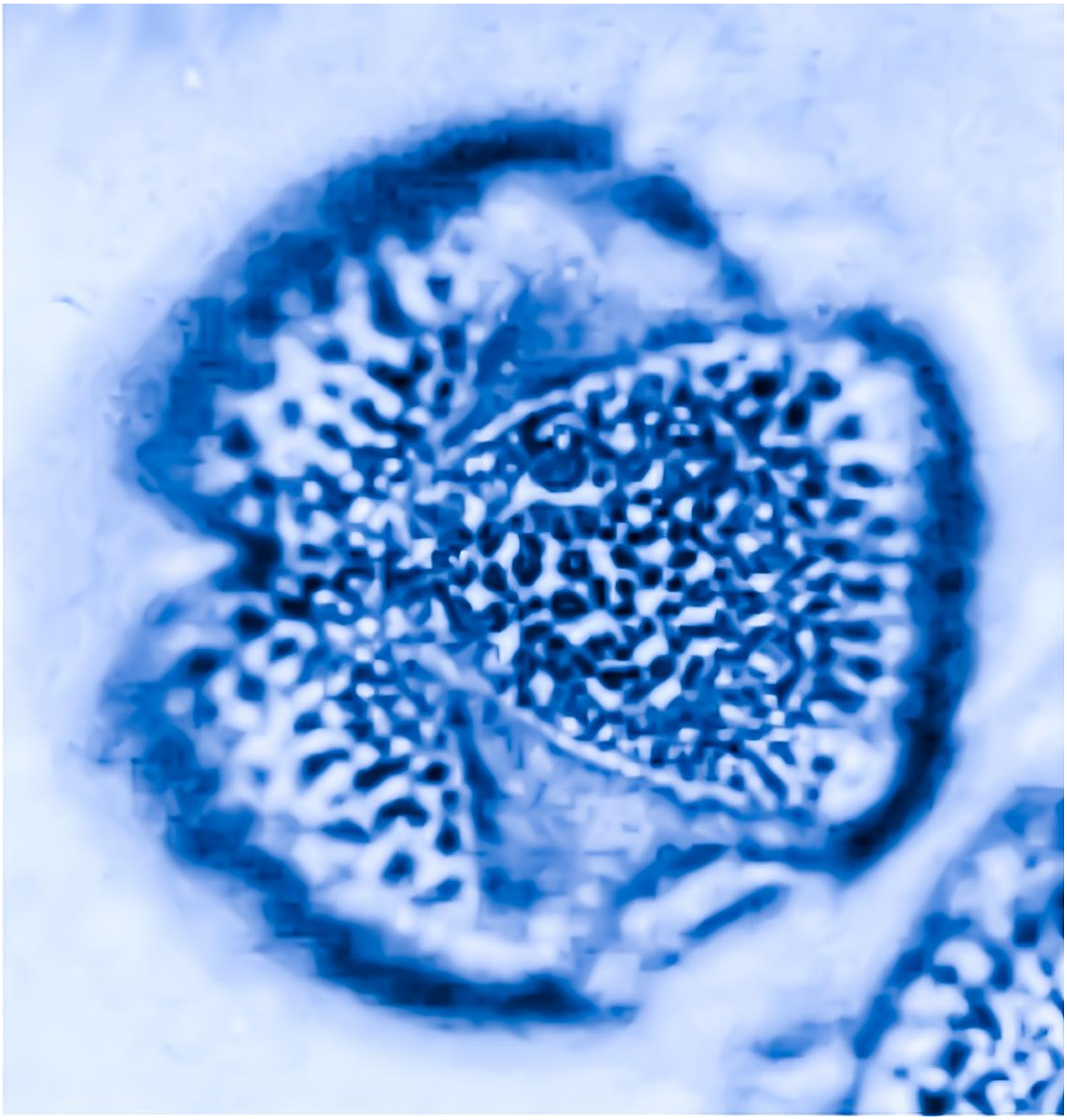

Supplement: Supplementary file 1 [file antibiotics-12-01197-s001.zip › antibiotics-2459539-supplementary.pdf]
